# Combined Anti-Biofilm Enzymes Strengthen the Eradicate Effect of *Vibrio parahaemolyticus* Biofilm: Mechanism on *cpsA-J* Expression and Application on Different Carriers

**DOI:** 10.3390/foods11091305

**Published:** 2022-04-29

**Authors:** Yuan Li, Ruyue Dong, Lei Ma, Yilin Qian, Zunying Liu

**Affiliations:** 1 College of Food Science and Engineering, Ocean University of China, Qingdao 266003, China; liyuan19930603@outlook.com (Y.L.); dry931857206@163.com (R.D.); malei@ouc.edu.cn (L.M.); qianyl92@outlook.com (Y.Q.); 2 Qingdao Engineering Research Center for Preservation Technology of Marine Foods, Qingdao 266003, China

**Keywords:** anti-biofilm enzymes, *Vibrio parahaemolyticus*, biofilm, exopolysaccharide

## Abstract

*Vibrio parahaemolyticus* is a human foodborne pathogen, and it can form a mature biofilm on food and food contact surfaces to enhance their resistance to antibacterial agents. In this study, the effect of anti-biofilm enzymes (combined lipase, cellulase and proteinase K) on the inhibition and eradication of pathogen biofilm was evaluated. The biofilm content of *V. parahaemolyticus* showed the highest level at the incubation time of 24 h, and the combined enzymes significantly inhibited the biofilm’s development. The biofilm’s inhibition and eradication rate at an incubation time of 24 h was 89.7% and 66.9%, respectively. The confocal laser scanning microscopic images confirmed that the microcolonies’ aggregation and the adhesion of biofilm were inhibited with the combined enzyme treatment. Furthermore, combined enzymes also decreased the concentration of exopolysaccharide (EPS) and disrupted the EPS matrix network, wherein the expression of the EPS-related gene, *cpsA-J*, was likewise suppressed. The combined enzymes showed an excellent inhibition effect of *V. parahaemolyticus* biofilm on different carriers, with the highest inhibition rate of 59.35% on nonrust steel plate. This study demonstrates that the combined enzyme of lipase, cellulase and proteinase K could be a novel candidate to overcome biofilm’s problem of foodborne pathogens in the food industry.

## 1. Introduction

*Vibrio parahaemolyticus* is a Gram-negative bacterium and a common pathogen in seafood. It forms biofilm during infection, which is an assemblage of self-produced polysaccharide, proteins and lipids, enveloping microbial surfaces [1]. The formation of biofilm can enhance resistance to adverse conditions and antibiotics, which plays an important role in the pathogenesis. As reported by Han et al. [2] and Almohamad et al. [3], biofilm causes more than 60% of foodborne outbreaks. The tolerance of biofilms to all antimicrobial agents is a thousand times higher than their planktonic counterparts. Therefore, it is difficult to eradicate biofilm with ordinary antibiotics and disinfectants [2].

Microscopic analyses have shown that the initial adhesion, formation of microcolonies and biofilm maturation were the processes of biofilm formation [4]. A major reason for food spoilage and transmission of foodborne pathogens is biofilm formation in which the high moisture content, microorganisms and nutrients present in the raw materials are aided. In the food industry, to mitigate undesirable biofilm effects, aggressive chemicals, such as sodium hydroxide or sodium hypochlorite, are often used. However, such approaches can corrode machinery and materials, negatively impacting on the environment [5,6]. Therefore, the development of an efficient approach that can control and remove bacterial biofilm is necessary. 

Anti-biofilm enzymes are considered innovative and environmentally friendly agents for biofilm control due to the fact that they can decompose extracellular matrix and promote biofilm detachment [7]. Studies have shown that biofilm cab be successfully destroyed by amylase and proteinase [8]. Cellulase was effective in partially inhibiting biomass and microcolony formation by *Pseudomonas aeruginosa* on glass surfaces [9]. α-Amylas could rapidly detach biofilms of *Bacillus subtilis S*8–18 as well as inhibit biofilm formation [10]. In recent years, several studies combined enzymes with other substances have been carried out for the prevention and treatment of biofilm, such as cetyltrimethylammonium bromide combined with either β-glucanase or α-amylase, which increased the removal rate of biofilm [11]. In addition, biofilm formation in wastewater treatment system could be effectively inhibited by the combination of vanillin with deoxyribonuclease or proteinase [12]. 

Anti-biofilm enzymes have shown an effective way to remove bacterial biofilm. Nevertheless, there is a lack of relevant understanding about the effects of enzymatic combinations on bacterial biofilms. The extracellular matrix of bacteria can basically be divided into polysaccharides, proteins and nucleic acids. Different degrading enzymes are selected according to the main components of the extracellular matrix of different bacteria. It has been reported that cellulase inhibits the formation of *Pseudomonas aeruginosa* biofilm. The effect of cellulase on the decomposition of exopolysaccharide (EPS) is supported by the decrease in the apparent molecular weight and the increase in the reducing sugar production. Cellulase was able to remove 94% of the *Pseudomonas fluorescens* biofilm formed on the surface of borosilicate glass [9]. Proteinase K could remove 33.7% of the cell membrane in reverse osmosis [3]. Microbial lipase has a wide range of action pH and temperature, high stability and activity. It can gradually hydrolyzes triglycerides into glycerol and fatty acids. Those three enzymes have been clarified to have a critical effect on biofilm removal. The objectives of the present study were to explore the effects of *V. parahaemolyticus* biofilm removal of combined enzymes (i.e., lipase, cellulase and proteinase K) by evaluating the eradication effect and exopolysaccharide disruption and to examine the inhibition effect of the combined enzymes on different carriers. The findings might contribute to the control of *V. parahaemolyticus* biofilm and promote the further application of anti-biofilm enzymes.

## 2. Materials and Methods

### 2.1. Bacterial Strain and Culture Conditions 

In this study, the bacterial strain used was *Vibrio parahaemolyticus* VIB461, which was provided by the Applied Marine Microbiology Laboratory of Ocean University of China (Qingdao, China). The *V. parahaemolyticus* was resuscitated from cryo stocks activated overnight in a 30 °C incubator on an agar plate (HBPM033, Qingdao Haibo Co., Ltd., Qingdao, China), then inoculated in tryptic soy broth (TSB with 3% NaCl), cultured at 30 °C with shaking (180 rpm) for 12 h. The colony number was measured by a spectrophotometer (UV2550, Shimadzu Co., Ltd., Kyoto, Japan) at OD_595nm_, the bacterial cell suspensions were adjusted to 1.0 × 10^6^ cfu/mL. A plate count was applied to validate the accuracy of the concentration of bacterial cell suspensions.

### 2.2. Preparation of Combined Enzymes

Lipase (20 U/mg, operation temperature: 15–45 °C, operation pH: 4.0–11.0), cellulase (3 U/mg, operation temperature: 15–55 °C, operation pH: 3.5–6.0) and proteinase K (30 U/mg, operation temperature: 15–65 °C, operation pH: 4.0–12.5) were purchased from Solarbio Co. (Beijing, China). The cellulase was dissolved in the buffer solution at pH 4.8. Lipase and proteinase K were dissolved in sterile water. Combinatorial enzymes consisted of 22 U/mL lipase, 3 U/mL cellulase and 45 U/mL proteinase K (final concentration) with a pH of 5.4. 

### 2.3. Analysis of the Biofilm Formation of V. parahaemolyticus 

The bacterial cell suspensions (1.0 × 10^6^ cfu/mL) with the volume of 200 μL were added to the 24-well microtiter plates with a lid (LY25, Greiner Co., Ltd., Stuttgart, Germany), and the plates were incubated in an incubator (SHP-2500, Jinghong Co., Ltd., Shanghai, China) at 30 °C with shaking (180 rpm) for 3, 6, 12, 24, 36 and 48 h. The amount of biofilm was measured using crystal violet staining according to the methods of Zhang et al. [13]. Each well was gently washed three times by sterile distilled water with 300 μL. The bacteria were fixed by dehydrated at 60 °C for 30 min, then 200 μL of 0.1% crystal violet solution were added to each well, followed by an incubation at ambient temperature for 15 min. The excess stain was removed by rinsing with sterile distilled water 3 times at 60 °C. The stain was released from adherent cells by adding 200 μL ethanol (95%). The value of OD_595nm_ measured by a multimode reader (Synergy H1, Bio-Tek Co., Ltd., Santa Clara, CA, USA) was used to describe the amount of biofilm. Three technical replicates and three biological replicates were conducted.

### 2.4. Analysis of the Biofilm Formation and Eradication of V. parahaemolyticus 

The 200 μL bacterial cell suspensions (1.0 × 10^6^ cfu/mL) and combined enzymes were added to 24-well microtiter plates with a lid (LY25, Greiner Co., Ltd., Stuttgart, Germany) in a ratio of 1:3 to make the final concentration of the enzyme solution as the test concentration. The microtiter plates were incubated at 30 °C with shaking (180 rpm) for 6, 24 and 48 h. Pipetting removed the broths carefully after incubation, and the cells that were planktonic and loosely attached were removed. The 1 mL sterile distilled water was used to wash the biofilms adhered onto the bottom and sides of the microtiter plate. The amount of biofilm was measured using a crystal violet staining method as described above. The biofilm formation rate (%) = (biofilm of control (OD_595nm_)-biofilm of treatment (OD_595nm_))/biofilm of control (OD_595nm_) × 100. The control group consisted of 20 μL bacterial cell suspensions (1.0 × 10^6^ cfu/mL) and a 60 μL buffer solution of pH 4.8, conducted on the same microtiter plates. Three technical replicates and three biological replicates were conducted.

For the biofilm eradication rate analysis, the *V. parahaemolyticus* cell suspensions (1.0 × 10^6^ cfu/mL) in microtiter plates were incubated at 30 °C with shaking (180 rpm) for 6, 24 and 48 h. The broths were removed, and the precipitates were washed with 1 mL sterile distilled water; then, they were added with 50 μL combined enzymes (1.1 mg/mL lipase, 1.0 mg/mL cellulase and 1.5 mg/mL proteinase K, final concentration). The plates were incubated at 37 °C for 4 h, and then the liquids were removed, and the biofilm contents were analyzed. The biofilm eradication rate (%) = (biofilm of control (OD_595nm_)-biofilm of treatment (OD_595nm_))/biofilm of control (OD_595nm_) × 100. The biofilm eradication effects of single enzymes (3.6 mg/mL lipase, 3.6 mg/mL cellulase and 3.6 mg/mL proteinase K) on *V. parahaemolyticus* were also evaluated at the incubation time of 24 h. 

### 2.5. Analysis of Exopolysaccharide (EPS) 

The extracellular EPS was detected according to the method of Xie et al. [14]. One milliliter of bacterial solution was taken from 24-well microtiter plates, centrifuged at 4000 rpm for 10 min at 4 °C. Afterwards, 3 mL sterilized normal saline was added into the cell pellets. After, the enzymatic hydrolysis, proteins were precipitated, and the supernatant was collected at 10,000 rpm for 30 min at 4 °C. To precipitate the polysaccharide, an equal volume of alcohol was added to the supernatant, and the whole solution was held at 20 °C for 1 h. The polysaccharide concentration was measured using the phenol sulphoacid method. The results of the EPS are expressed as the values of OD_490nm_.

### 2.6. Observation of Biofilm by CLSM and SEM

Twenty milliliters of bacterial cell suspensions and the combined enzymes were added to a Petri dish (diameter of 60 mm) in a ratio of 1:3, and the final concentration of the enzyme solution was the same as the test concentration. The Petri dish was incubated at 30 ℃ for 24 h. To remove the planktonic cells on the surface, PBS was used to wash the Petri dish three times, gently. Then, 5 mL of fluoresce inisothiocyanate (FITC) Con-A (Sigma–Aldrich, Gillingham, UK) was added and maintained for 20 min at 4 °C in the dark. Then, the Petri dish was gently washed three times with PBS again. The biofilms were observed under a confocal laser scanning microscope (CLSM, ZEISS, Oberkochen, Germany) in the excitation wavelength of 488 nm. 

The bacterial cell suspensions (1.0 × 10^6^ cfu/mL) and combined enzymes were added to the test tube in a ratio of 1:3, and the final concentration of the enzyme solution was set as the test concentration. Then, a sterilized glass sheet (2 cm × 1 cm × 2 mm) was put into the test tube. The control group was added into the same volume of buffer solution. The test tubes were incubated at 30 °C with shaking (180 rpm) for 24 h. The glass sheet was prepared for scanning electron microscopy (SEM) as previously described. 

### 2.7. Analysis of cpsA-J Gene Expression

The bacteria cultured in both combined enzymes and sterile distilled water for 24 h were harvested for RNA extraction. Total RNA was extracted according to the manufacturer’s instructions (Bacteria Total RNA Isolation Kit Sangon Biotech, Shanghai, China). RNA quantity was determined using a NanoDrop spectrophotometer (Nano-200, Hangzhou Austrian Sheng Instrument Co., Ltd., Hangzhou, China). A Bio-Rad CFX Connect System (Bio-Rad Laboratories, Inc., Hercules, CA, USA) was used to performed RT-qPCR amplifications with 3 biological replicates. The housekeeping gene 16S rRNA was used as a control for normalization, and the *cpsA-J* and 16s rRNA primer sequences are shown as follows: 16S rRNA, Forward (5’-3’): CGTAGGTGGCAAGCGTTGTCC; Reverse (5’-3’): CGCCTTCGCCACTGGTGTTC. *cpsA-J*, Forward (5’-3’): AAGCAGAGCGTGAAGCACTAGC; Reverse (5’-3’): TTCAACGTCGATACCGTCTTGAGC. Relative expression of the gene = 2^-^^△△CT^, △CT = CT of target gene-CT of 16S rRNA; △△CT = △CT of the treated sample-CT of the control sample.

### 2.8. Preparation and Characterization of V. parahaemolyticus Biofilm on Different Carriers

The sterilized nonrust steel sheet and the glass sheet with a size of 2 × 1 cm were immersed into the V. parahaemolyticus suspensions (1.0 × 10^6^ cfu/mL), prepared as described in Section 2.3, and incubated at 30 °C with shaking (180 rpm) for 24 h. The carriers were taken out and put into sterilized normal saline for ultrasonic treatment for 10 min, and the number of bacteria was calculated.

The sterilized nonrust steel sheet and the glass sheet with a size of 2 × 1 cm were immersed into the TSB medium. The V. parahaemolyticus suspension (1.0 × 10^8^ cfu/mL) was added with a 1% inoculation amount, and the combined enzyme was added with a 3% inoculation amount. Samples were cultured at 180 r/min for 24 h at 30 °C. Then, the barriers were taken out. The biofilm formation and inhibition degrees were measured and calculated according to Section 2.3 and Section 2.4.

The EPS changes on different carriers were conducted according to Section 2.5.

### 2.9. Statistical Analysis

Statistical analysis was performed using the SPSS 20.0 statistics software. A *p* < 0.05 was considered significant. All graphical evaluations were made using GraphPad Prism 6.0.

## 3. Results and Discussion 

### 3.1. The Dynamic Development of V. parahaemolyticus Biofilm Formation

**Figure 1:**
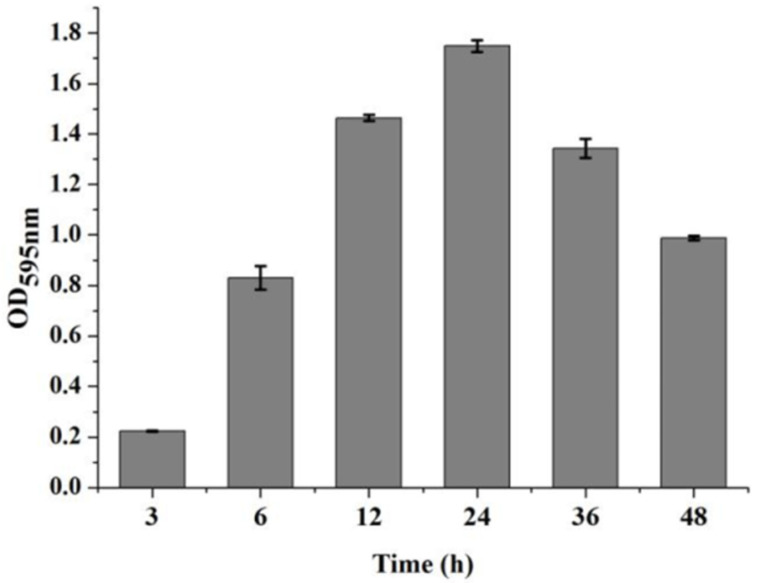
The dynamic development of *V. parahaemolyticus* biofilm formation incubated in 24-well microplates. Three technical replicates and three biological replicates were conducted.

In order to evaluate the effects of combined enzymes on biofilm formation and eradication, the dynamic development of *V. parahaemolyticus* biofilm formation was first detected. The results indicated that the biomass of *V. parahaemolyticus* biofilm increased with the increase in incubation time and reached the maximum value at the incubation time of 24 h. Then, the biomass of the film decreased when the incubation time was longer than 24 h. Therefore, the initial phase of the biofilm formation was from 2 to 6 h, the mature phase was from 6 to 24 h, and the dispersal phase was from 24 to 48 h (Figure 1). 

### 3.2. Effects of Combined Enzymes on the Biofilm Formation and Eradication of V. parahaemolyticus

**Figure 2:**
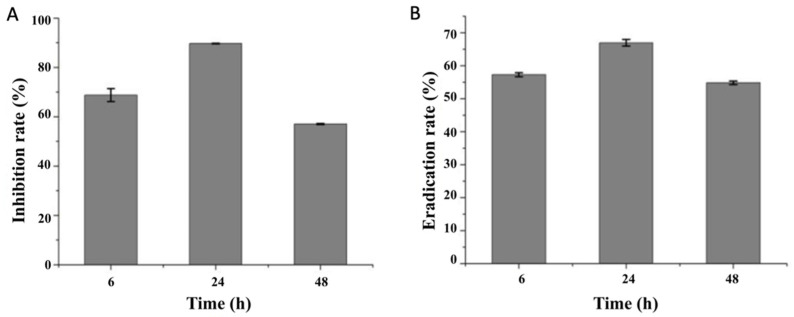
Effects of combined enzymes on *V. parahaemolyticus* biofilm: (**A**) formation rate; (**B**) eradication rate. Three technical replicates and three biological replicates were conducted.

The results of the biofilm formation rate showed that the biofilm formation of *V. parahaemolyticus* was significantly inhibited with anti-biofilm enzyme treatments (Figure 2A). As shown in Table 1, among three enzymes, cellulase showed the highest biofilm inhibition rate at an incubation time of 24 h, and the biofilm inhibition rate of the combined enzymes was significantly enhanced compared with the single enzymes (*p* < 0.05). 

Because the biofilm extracellular polymeric substances might contain proteins, DNA and polysaccharides, the use of a combination of anti-biofilm enzymes or an anti-biofilm enzyme with other substances could be a more effective way to control and remove biofilms. Previous studies have shown that cellulase combined with pronase could remove 94% of *P. aeruginosa* biofilm formed on borosilicate glass surfaces [15]. The combination of DNase I and AlgL enzymes showed a better effect in destroying the biofilm structure of *Enterococcus faecalis* and *Enterococcus faecium.* Combining hydrodynamic and enzymatic treatments led to an increase of 80% in biofilm mass removal compared to the enzymatic treatment alone [16]. 

In the different phases of the biofilm formation, the inhibition effects of combined enzymes were different, with inhibition rates of 68.79%, 89.65% and 57.04% at the incubation times of 6, 24 and 48 h, respectively. The combined enzymes also provided an effective way to remove the formed biofilm, and the biofilm eradication rate at the incubation times of 6, 24 and 48 h was 57.62%, 67.65% and 54.87%, respectively (Figure 2B). 

The biofilm removal effect of combined enzymes could be affected if interaction among enzymes exists. Özmen [17] evaluated the detergent compatibility of proteinase and cellulase, suggesting that both of them have strong excellent compatibility. Naganthran et al. [18] clarified that the compatibilities of lipase and proteinase were excellent. Eisazadeh et al. [19] proved that the activity of lipase and/or cellulase was not affected during interaction. Moreover, some kinds of cellulases have been clarified to be proteinase resistant [20]. Those previous studies and our current research all indicate that the overall effect of the combined enzymes on biofilm was not significantly affected by their interaction.

Our study indicated that proteinase K, lipase and cellulase inhibited biofilm formation of *V. parahaemolyticus* by 58.6%, 60.2% and 80.9%, respectively (Table 1). The combined proteinase K, lipase and cellulase showed better effects in controlling biofilm formation of *V. parahaemolyticus* compared to an arbitrary one, which might be attributed to the biofilm matrix’s broad dispersal and multi-dimensional degradation after combined enzyme treatment. These results indicate that the use of combined enzymes is an effective way to control and remove the biofilm of *V. parahaemolyticus*. On the other hand, the volume of samples in this study was small. Less dense biofilms might exaggerate the efficacy, which may contribute to the reduced biofilm observed. In future applications, an experiment on biofilm inhibition in larger volumes is needed.

**Table 1:** Effects of combined enzymes on the biofilm eradication of *V. parahaemolyticus* incubated in 24-well microplates for 24 h.

Enzymes	Biofilm Inhibition Rate (%)
Proteinase K Lipase Cellulase Combined enzymes	58.6 ± 0.89 ^a^ 60.2 ± 1.84 ^a^ 80.9 ± 0.67 ^b^ 89.7 ± 0.16 ^c^

Mean values with different letters differ significantly at *p* < 0.05.

### 3.3. The CLSM Images of the Biofilm Structure of V. parahaemolyticus

**Figure 3:**
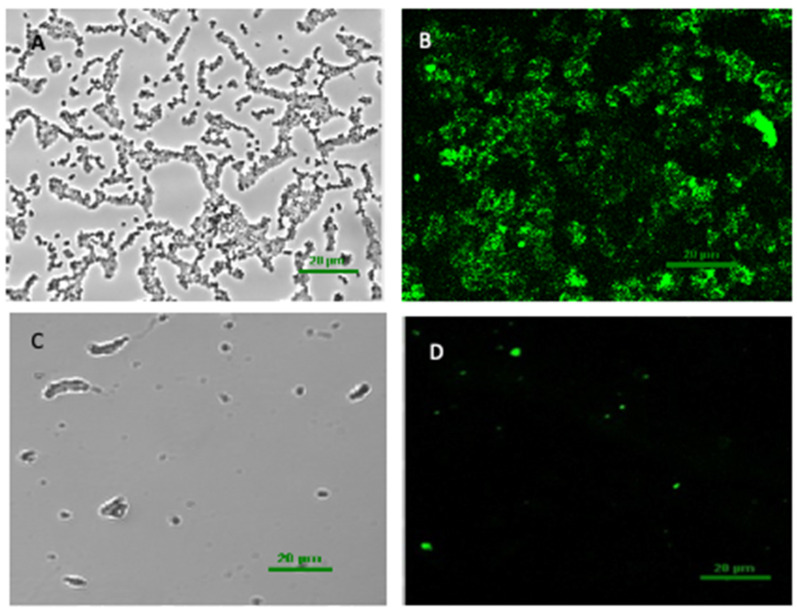
The representative confocal laser scanning microscope (CLSM) images of biofilm structure of *V. parahaemolyticus* incubated in 24-well microplates for 24 h. Control: (**A**,**C**) (addition of FITC); combined enzyme treatment: (**B**,**D**) (addition of FITC). The green color in (**B**,**D**) demonstrate the distribution of exopolysaccharide (EPS).

Confocal laser scanning microscopy (CLSM) was used to observe the biofilm to further understand the changes in biofilm structure. The CLSM images clearly showed that in the untreated biofilm, the volume was relatively larger, the distribution of polysaccharide was much denser, and the biofilm was thicker (Figure 3A,B). After the combined enzyme treatment, the extracellular polysaccharide content reduced significantly, because the green fluorescence intensity significantly decreased (Figure 3C,D). The combined enzymes could effectively decrease the biofilm matrix of *V. parahaemolyticus*. This result showed that anti-biofilm enzymes removing biofilm of *V. parahaemolyticus* is based on the biofilm matrix degradation. Among the enzymes, cellulase showed a better effect at removing the biofilm, indicating that the biofilm matrix components of *V. parahaemolyticus* might contain a large amount of exopolysaccharide. In this regard, the appropriate strategy for biofilm removal is using an enzyme that can effectively degrade extracellular polymeric substances produced by the pathogen. Previous research has shown that the biofilm formed by *E. faecalis* contained a great deal of extracellular DNA but a low level of extracellular polysaccharides [21], and accordingly DNase I showed high removal efficiency towards *E. faecalis* biofilm. Therefore, to extend the applicability of anti-biofilm enzymes, further studies are required to determine the effect of the combined enzymes on mixed-species biofilms with different extracellular polymeric substances, which are likely to be present in food processing environments. 

### 3.4. The SEM Images of the Biofilm Structure of V. parahaemolyticus

**Figure 4:**
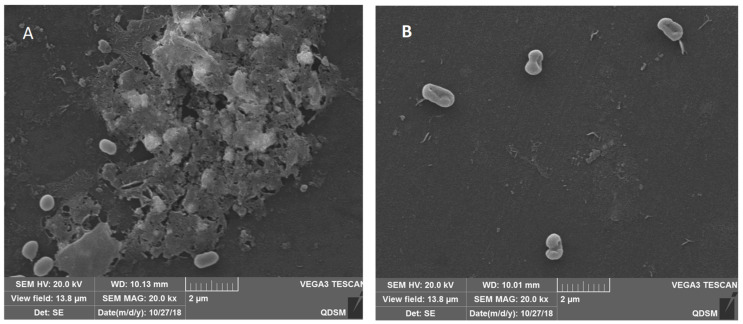
The representative scanning electron microscopy (SEM) images of biofilm structures of *V. parahaemolyticus* incubated in 24-well microplates for 24 h: (**A**) control; (**B**) combined enzyme treatment (20,000×).

To gain further insight into the changes in the structure of biofilm, scanning electron microscope (SEM) images of *V. parahaemolyticus* biofilm treated with combined enzymes were taken. The SEM images reconfirmed the results of the CLSM. The SEM images showed that the bacteria were coated with a biofilm matrix (Figure 4A), and only a few bacteria adhered to the board after the combined enzyme treatment (Figure 4B). These images revealed that the biofilm matrix was disrupted, and most of the biofilm cells were detached from the biofilm matrix after combined enzyme treatment. The biofilm cells could obstruct the penetration of antimicrobials and the access to the deep positioned cells because of their compact and complex structure; thus, the biofilms were highly resistant to stress conditions [22]. The matrix that forms the basis of the three-dimensional structure constituted the biofilm and accounted for more than 90% of the dry mass of a biofilm. Consequently, degradation of the matrix is an effective method for deracinating microbial biofilm [23]. The use of enzymes is a direct anti-biofilm approach. Previous studies have shown that some single enzymes can effectively inhibit bacterial biofilm. Loiselle et al. reported that the enzyme cellulase could inhibit *P. aeruginosa* biofilm formation [9]. In another study, α-amylase reduced *S. aureus* biofilm by 79% [10].

### 3.5. Effects of Combined Enzymes on EPS Concentration and cpsA-J mRNA Relative Expression of V. parahaemolyticus 

**Figure 5:**
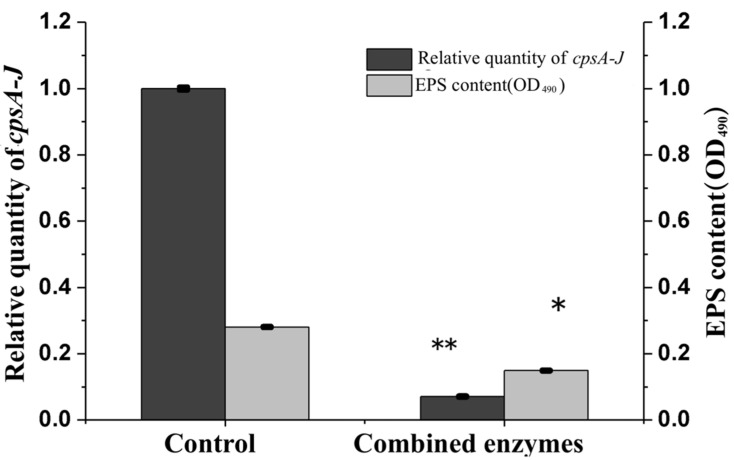
Effects of combined enzymes on exopolysaccharide (EPS) concentration and *cpsA-J* mRNA’s relative expression of *V. parahaemolyticus* incubated in 24-well microplates. * *p* < 0.05, ** *p* < 0.01. Three technical replicates and three biological replicates were conducted.

The genes responsible for EPS production in *V. parahaemolyticus* are located on the VPA1403-1412 (*cpsA-J*) operon. To further explore the effect of combined enzymes on the biofilm formation of *V. parahaemolyticus*, the EPS concentration and *cpsA-J* mRNA relative expression of *V. parahaemolyticus* were analyzed. The results indicated that the EPS concentration of *V. parahaemolyticus* with combined enzyme treatment was apparently lower than that of the control at an incubation time of 24 h (Figure 5, *p* < 0.05), and the same trend was observed in *cpsA-J* mRNA’s relative expression, indicating that both EPS production and EPS biosynthesis were regulated by combined enzymes during the incubation of *V. parahaemolyticus.*

The biofilm formation of *V. parahaemolyticus* is a complex and differentiated dynamic process involving cell–surface and cell–cell interactions [24], which can be inhibited and removed by different methods. It is reported that acidic electrolyzed water can inhibit *V. parahaemolyticus* biofilm formation through triggering EPS disruption by the deformations of the carbohydrate C–O–C bond and the aromatic rings in the amino acids [25]. Cu nanoparticles inhibited more than 60% *V. parahaemolyticus* biofilm formation by inducing biofilm matrix morphological changes and reducing extracellular polysaccharide production. Chitosan also inhibited the biofilm formation by 70.98% and 68.37% at the minimum inhibition concentration (MIC) and ½ MIC concentration [14]. In this study, the combined enzymes effectively inhibited the expression of *cpsA-J* mRNA in *V. parahaemolyticus*; then, the biofilm formation was regulated by controlling the production of EPS through the expression of *cpsA-J* mRNA.

### 3.6. Inhibition Effect of the Combined Enzymes on Different Carriers

The biofilm formation of *V. parahaemolyticus* usually occurs in the instruments during the process of food production or in glassware containing seafood or pickled products, which not only pollutes the equipment but also often causes cross-infection and food poisoning. Therefore, how to effectively prevent these equipment or vessels from being polluted by *V. parahaemolyticus* is a particularly important issue. Exploring the inhibition effect of the optimized composite enzyme on *V. parahaemolyticus* biofilm on different material carriers could provide a theoretical basis for the commercial and targeted application of the combined enzymes.

**Figure 6:**
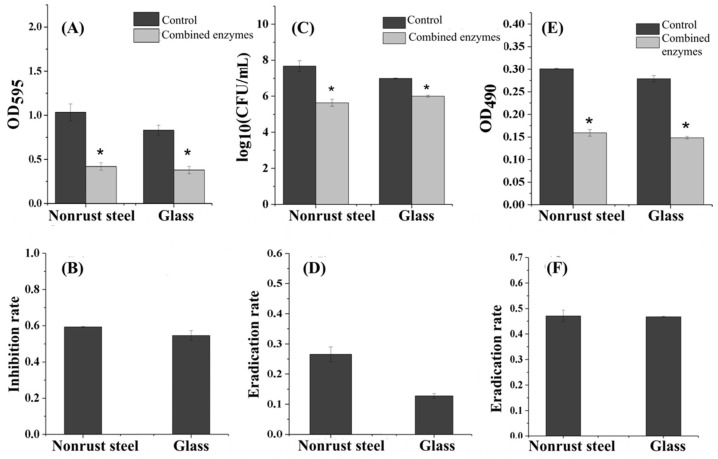
Effect of combined enzymes for biofilm eradication on different carriers: (**A**,**B**) inhibition effect; (**C**,**D**) the number of bacteria; (**E**,**F**) the content of extracellular polysaccharide. Three technical replicates and three biological replicates were conducted. In the figure, * represents a significant difference with *p* < 0.05.

The inhibition effect of combined enzymes on biofilm on different carriers are shown in Figure 6A,B. The forming ability of *V. parahaemolyticus* biofilm on different carriers was different. The forming ability on a nonrust steel sheet was stronger than on a glass sheet. The complex enzyme, composed of cellulase, lipase and proteinase, had significant inhibition effects on the biofilm of *V. parahaemolyticus* on nonrust steel and glass, and the inhibition rates were 59.35 ± 0.13% and 50.09 ± 2.64%, respectively. The effects of combined enzymes on the numbers of bacteria on different carriers are shown in Figure 6C,D. On both carriers, the numbers of bacteria in combined enzyme-treated-groups were lower than in the control groups, while the reduction rate on nonrust steel were higher. Studies have shown that there is a significant correlation between the formation of biofilm and the embedded bacteria in biofilm. A study has shown that the number of bacteria in *S. aureus* biofilm decreased with the increase in the benzonase enzyme concentration, and the thickness of the biofilm also decreased [26]. They found that 0.70 mM carvacrol could reduce the adhesion of *Salmonella* on the surface of nonrust steel by 2.0 lg (cfu/mL). Therefore, it was concluded that carvacrol led to a reduction in bacterial adhesion and the formation of weak biofilm. Proteinase K had no significant effect on the biofilm attached by *Listeria* on glass, while Nguyen et al. [15] found that proteinase K could significantly reduce the attachment of *Listeria* on nonrust steel.

The contents of extracellular polysaccharide in biofilm on different carriers are shown in Figure 6E,F. Both carriers showed similar eradication rates. Previous studies have shown that inhibiting the attachment of bacteria to nonrust steel can inhibit the formation of bacterial biofilm on nonrust steel. In this experiment, the combined enzyme could also reduce the attachment of *V. parahaemolyticus* by 2.3 lg (cfu/mL) on the surface of nonrust steel, significantly inhibiting the formation of biofilm of *V. parahaemolyticus* on the nonrust steel. However, the types of bacteria impacted the final effects based on the previous research results.

### 3.7. SEM Images of Combined Enzymes-Inoculated Carriers

**Figure 7:**
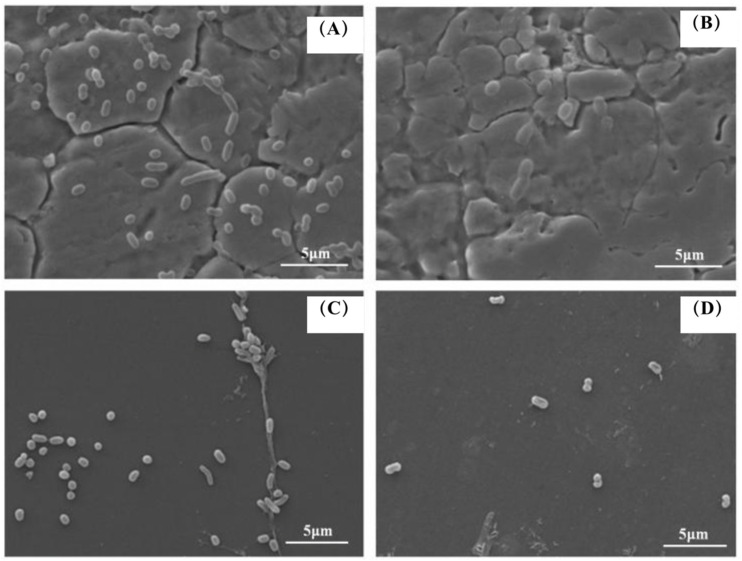
Effect of combined enzymes on the structure of biofilm on different carriers (representative images): (**A**) control group on nonrust steel; (**B**) combined enzymes on nonrust steel; (**C**) control group on glass; (**D**) combined enzymes on glass.

The typical morphology of biofilms on different carriers is shown in Figure 7. Through a scanning electron microscope, it was observed that the state of bacteria adhering to nonrust steel and the inhibition effect of the compound enzyme showed significant differences. As shown in Figure 7, in the control group, a large quantity of bacteria could adhere to the nonrust steel, leading to biofilm formation. After treatment with the combined enzymes, only individual bacteria adhered to the nonrust steel plate without forming biofilm. In the control group, many bacteria were adhered to the glass. After treatment with combined enzymes, only a few bacteria adhered to the nonrust steel plate, with little biofilm formation. There was no significant difference in the ability of bacteria adhering to the nonrust steel and glass, which also confirms the previous conclusion. Cai et al. [27] reported that the acidic electrolytic water can effectively inhibit the formation of fluorescent *Pseudomonas* biofilm. The results of SEM images showed that the fluorescent *Pseudomonas* biofilm treated by acidic electrolytic water was separated and decomposed into small extracellular matrix aggregates, and the amount of extracellular matrix and bacterial adhesion was significantly reduced compared with the untreated group. 

Through scanning electron microscopy, Lekbach et al. [28] observed that the biofilm of *P. aeruginosa* on nonrust steel treated with *Salvia miltiorrhiza* extract changed from the original tight network structure to loosen small aggregates, and the cell morphology changed accompanied by the extravasation of internal solute. It was found that the *S. miltiorrhiza* extract might inhibit the biofilm formation of *P. aeruginosa* on nonrust steel by acting on the cell membrane of bacteria. SEM study indicated that compared with the control group, 200 μg/mL proteinase K could completely remove the biofilm of *Listeria* on nonrust steel [15]. Although the composite enzyme containing proteinase K could effectively reduce the adhesion of *V. parahaemolyticus* and the formation of extracellular aggregates on nonrust steel, it could not completely remove the biofilm, which may be due to the different types of extracellular matrixes of different bacteria biofilms. The content of various substrates was also different, which suggested that the protein content in the substrate of *V. parahaemolyticus* biofilm might be less than that of *Listeria*. Previous studies found that DNase I inhibited and dispersed *S. aureus* biofilm, while no effect was observed on *Staphylococcus epidermidis* biofilm [26]. Therefore, the combined enzymes used in this experiment could effectively reduce the adhesion and breeding of *V. parahaemolyticus* on food processing utensils and further improve food safety. However, those experiments were conducted in vitro under laboratory conditions. When transfer into practical applications, validation experiments should be performed.

## 4. Conclusions

In conclusion, this study evaluated the potential of anti-biofilm enzymes (combined lipase, cellulase and proteinase K) as an anti-biofilm candidate against foodborne pathogen *V. parahaemolyticus* VIB461. The combined enzymes could effectively inhibit all stages of biofilm formation and remove the mature biofilm. More importantly, the combined enzymes effectively regulated the exopolysaccharide matrix production and biosynthesis of *V. parahaemolyticus.* Although some foods are preserved under room temperature, their preservation temperature could be different. For example, some muscle foods are preserved under refrigerated condition. Under lower temperatures, the activity of enzymes may be affected. Since the experiments in this research were conducted at 30 °C, further research on the effect of temperature in combined enzymes towards the biofilm inhibition should be conducted for a broader application in the food preservation. With theoretical basis from this research, we believe that the hybrid application of lipase, cellulase and proteinase K could be a promising anti-biofilm strategy for *V. parahaemolyticus* and food pathogens. These results cannot be generalized to all *V. parahaemolyticus* strains but can be taken to suggest that this approach might be effective against some strains of this pathogen.

## Data Availability

Data is contained within the article.
